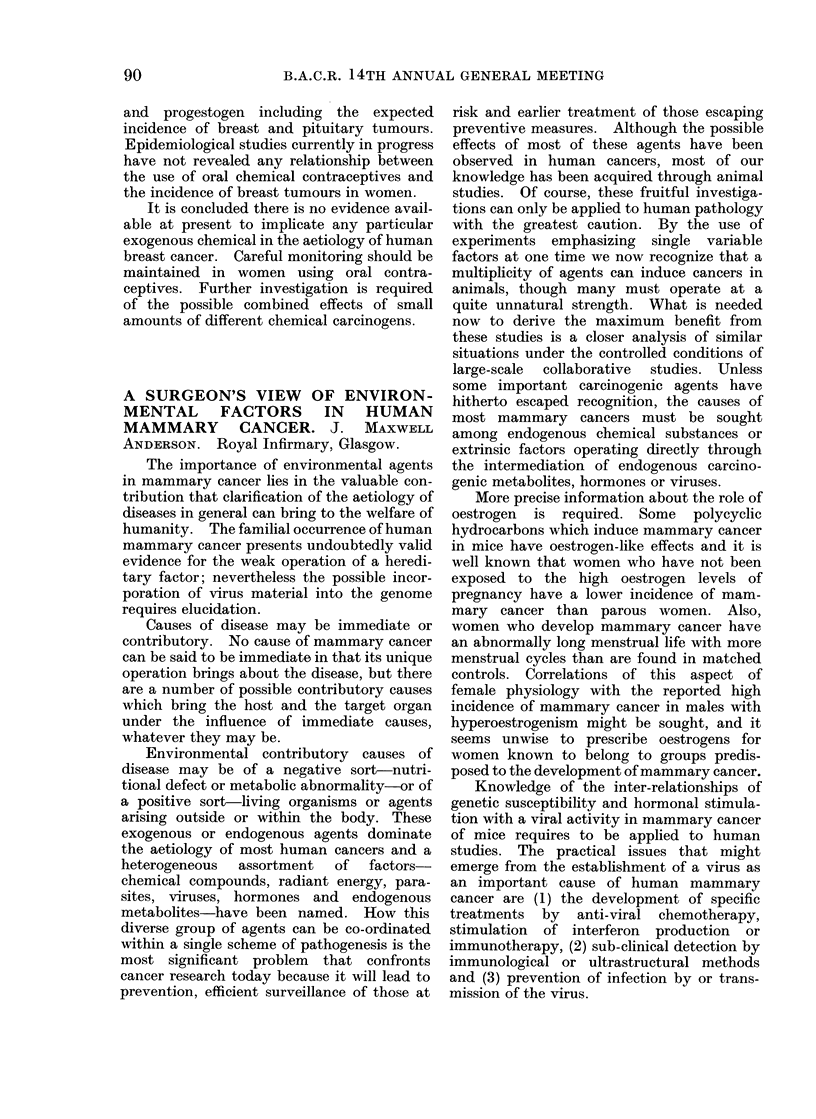# A surgeon's view of environmental factors in human mammary cancer.

**DOI:** 10.1038/bjc.1973.117

**Published:** 1973-07

**Authors:** J. M. Anderson


**A SURGEON'S VIEW OF ENVIRONMENTAL FACTORS IN HUMAN MAMMARY CANCER.** J. MAXWELL ANDERSON. Royal Infirmary, Glasgow.

The importance of environmental agents in mammary cancer lies in the valuable contribution that clarification of the aetiology of diseases in general can bring to the welfare of humanity. The familial occurrence of human mammary cancer presents undoubtedly valid evidence for the weak operation of a hereditary factor; nevertheless the possible incorporation of virus material into the genome requires elucidation.

Causes of disease may be immediate or contributory. No cause of mammary cancer can be said to be immediate in that its unique operation brings about the disease, but there are a number of possible contributory causes which bring the host and the target organ under the influence of immediate causes, whatever they may be.

Environmental contributory causes of disease may be of a negative sort—nutritional defect or metabolic abnormality—or of a positive sort—living organisms or agents arising outside or within the body. These exogenous or endogenous agents dominate the aetiology of most human cancers and a heterogeneous assortment of factors—chemical compounds, radiant energy, parasites, viruses, hormones and endogenous metabolites—have been named. How this diverse group of agents can be co-ordinated within a single scheme of pathogenesis is the most significant problem that confronts cancer research today because it will lead to prevention, efficient surveillance of those at risk and earlier treatment of those escaping preventive measures. Although the possible effects of most of these agents have been observed in human cancers, most of our knowledge has been acquired through animal studies. Of course, these fruitful investigations can only be applied to human pathology with the greatest caution. By the use of experiments emphasizing single variable factors at one time we now recognize that a multiplicity of agents can induce cancers in animals, though many must operate at a quite unnatural strength. What is needed now to derive the maximum benefit from these studies is a closer analysis of similar situations under the controlled conditions of large-scale collaborative studies. Unless some important carcinogenic agents have hitherto escaped recognition, the causes of most mammary cancers must be sought among endogenous chemical substances or extrinsic factors operating directly through the intermediation of endogenous carcinogenic metabolites, hormones or viruses.

More precise information about the role of oestrogen is required. Some polycyclic hydrocarbons which induce mammary cancer in mice have oestrogen-like effects and it is well known that women who have not been exposed to the high oestrogen levels of pregnancy have a lower incidence of mammary cancer than parous women. Also, women who develop mammary cancer have an abnormally long menstrual life with more menstrual cycles than are found in matched controls. Correlations of this aspect of female physiology with the reported high incidence of mammary cancer in males with hyperoestrogenism might be sought, and it seems unwise to prescribe oestrogens for women known to belong to groups predisposed to the development of mammary cancer.

Knowledge of the inter-relationships of genetic susceptibility and hormonal stimulation with a viral activity in mammary cancer of mice requires to be applied to human studies. The practical issues that might emerge from the establishment of a virus as an important cause of human mammary cancer are (1) the development of specific treatments by anti-viral chemotherapy, stimulation of interferon production or immunotherapy, (2) sub-clinical detection by immunological or ultrastructural methods and (3) prevention of infection by or transmission of the virus.